# Supplementary material for: KIF2A Upregulates PI3K/AKT Signaling through Polo-like Kinase 1 (PLK1) to Affect the Proliferation and Apoptosis Levels of Eriocheir sinensis Spermatogenic Cells
Source: Biology (Basel). 2024 Feb 27;13(3):149. doi: 10.3390/biology13030149 (PMC10968087; doi:10.3390/biology13030149)
Supplement: Supplementary file 1 [file biology-13-00149-s001.zip › biology-2828173-supplementary.pdf]

Supplementary Materials

```
   1 atg gcc agg ggt tcc ggc cgc ctt ctg agg tcc tcc cgc aca cac agc aac gtg ccc gcc
   1  M   A   R   G   S   G   R   L   L   R   S   S   R   T   H   S   N   V   P   A
  61 gcc gcc cac cac cct ctc aga cct cca tcc gcc cac aca tcc cac ttg ccc cgc ccg ccc
  21  A   A   H   H   P   L   R   P   P   S   A   H   T   S   H   L   P   R   P   P
 121 ggg cac cag gtg gcc cga tca cag tcc gcc tcc aga ctc tcc atg tac acg cgg cag tcc
  41  G   H   Q   V   A   R   S   Q   S   A   S   R   L   S   M   Y   T   R   Q   S
 181 caa gtc cct cag tct gct tcg act ccg gcc aca agg agc aaa gat gcc tcc aga ccc cgc
  61  Q   V   P   Q   S   A   S   T   P   A   T   R   S   K   D   A   S   R   P   R
 241 cca agc tac ctg gtc aag ccg ccc cag aat gga gag atc cgc tcc aac cta cag aac aag
  81  P   S   Y   L   V   K   P   P   Q   N   G   E   I   R   S   N   L   Q   N   K
 301 atc ggg cag tcc cag gcc agg tcg aca gaa aac atc tca acc tcc act gtc agc aag gga
 101  I   G   Q   S   Q   A   R   S   T   E   N   I   S   T   S   T   V   S   K   G
 361 tcc gtg cct gcc tcc atc ccg ccc gca gcc cgc cgc cgc agc aat gtg gtg aag gag gta
 121  S   V   P   A   S   I   P   P   A   A   R   R   R   S   N   V   V   K   E   V
 421 gat cgc atc cag aag aag agg gag gag cgg agg aag cag cag gcg gag aag aag gag gag
 141  D   R   I   Q   K   K   R   E   E   R   R   K   Q   Q   A   E   K   K   E   E
 481 aag gag gca ctc atg aat ctg gac ccc ggc aac ccg cag tgg gag ttc ctt aac atg atc
 161  K   E   A   L   M   N   L   D   P   G   N   P   Q   W   E   F   L   N   M   I
 541 aga gaa ttc agg tca caa cta gag ttc cgc aca ctg aaa gat gga gac ccg ctg gag gag
 181  R   E   F   R   S   Q   L   E   F   R   T   L   K   D   G   D   P   L   E   E
 601 cac cag att acc gtg gca gtc aga aaa aga ccc ctg aat aaa aaa gag aat agt aag cgc
 201  H   Q   I   T   V   A   V   R   K   R   P   L   N   K   K   E   N   S   K   R
 661 gag att gac gtg atc acc atc cct aag agg aac acc cta tac gtc cac gag ccc cgc acc
 221  E   I   D   V   I   T   I   P   K   R   N   T   L   Y   V   H   E   P   R   T
 721 aaa gtc gac ctc acc aag tac ctc gag aac cag aac ttc cgc ttt gac tac gcc ttc gac
 241  K   V   D   L   T   K   Y   L   E   N   Q   N   F   R   F   D   Y   A   F   D
 781 gaa acc tgt aac aac gaa ctc gtc tac aaa tac aca gcg aga cct cta gtt cag acc atc
 261  E   T   C   N   N   E   L   V   Y   K   Y   T   A   R   P   L   V   Q   T   I
 841 ttt gag ggt ggc atg gca acc tgt ttt gcc tac ggt cag acc ggc tcg ggc aag acg cac
 281  F   E   G   G   M   A   T   C   F   A   Y   G   Q   T   G   S   G   K   T   H
 901 acc atg ggg ggt gac ttc cag ggc aag aac cag gac tgt gca aag gga att tat gct atg
 301  T   M   G   G   D   F   Q   G   K   N   Q   D   C   A   K   G   I   Y   A   M
 961 gtg gcc aaa gac gtg ttt atc tac att aag aac cca aag tat aga aat ctt aac cta caa
 321  V   A   K   D   V   F   I   Y   I   K   N   P   K   Y   R   N   L   N   L   Q
1021 atc tct gct agc ttt ttt gaa att tat ggt ggt aag gtt ttt gat ctg ctc aac aac aag
 341  I   S   A   S   F   F   E   I   Y   G   G   K   V   F   D   L   L   N   N   K
1081 acg aag ctt cgg gtg ctg gag gac ggg aag aac gtg gtg cag gtg gtg ggg ctg cag gag
 361  T   K   L   R   V   L   E   D   G   K   N   V   V   Q   V   V   G   L   Q   E
1141 agg gca tgc gag agc gtg gac gat gtg ctc cgc ctc atc agt ctc ggc tcc caa gtg cgc
 381  R   A   C   E   S   V   D   D   V   L   R   L   I   S   L   G   S   Q   V   R
1201 acc tcc ggc caa acc acc gcc aac aac cag tcg tcg cgc tcc cat gct gtc ttc cag atc
 401  T   S   G   Q   T   T   A   N   N   Q   S   S   R   S   H   A   V   F   Q   I
1261 att ttg aga aac ttg gat aga ata gaa aag caa ggc gac aag gcg tat aaa ctc cac ggc
 421  I   L   R   N   L   D   R   I   E   K   Q   G   D   K   A   Y   K   L   H   G
1321 aag ttc tct ctc att gac ttg gcg ggc aat gag agg gga gca gac acc tcc agc gcc aac
 441  K   F   S   L   I   D   L   A   G   N   E   R   G   A   D   T   S   S   A   N
1381 agg cag acc cgc atg gaa ggg gca gag atc aac aag tcc ctc ctg gcc ctg aag gag tgc
 461  R   Q   T   R   M   E   G   A   E   I   N   K   S   L   L   A   L   K   E   C
1441 atc cgt gcg ctg ggc cgc aaa gga gct cac ctt ccc ttc agg gct tcc aag ctg acg cag
 481  I   R   A   L   G   R   K   G   A   H   L   P   F   R   A   S   K   L   T   Q
1501 gtg ctc cga gac tcc ttc atc ggg gac aag agc aag acc tgc atg att gcc atg atc tcc
 501  V   L   R   D   S   F   I   G   D   K   S   K   T   C   M   I   A   M   I   S
1561 ccc ggc atg aac tgc tgt gaa cac acg tta aac acc ctc agg tat gca gac agg gta aag
 521  P   G   M   N   C   C   E   H   T   L   N   T   L   R   Y   A   D   R   V   K
1621 gag ttg ggt ctc tct gaa agc act gag gac aat tac aag tcc acg cca ata gag aag gaa
 541  E   L   G   L   S   E   S   T   E   D   N   Y   K   S   T   P   I   E   K   E
1681 cct gac gag aat ggc acc aac aat gtt gac tac tcc act ctc tct aac ctc aat gaa aat
 561  P   D   E   N   G   T   N   N   V   D   Y   S   T   L   S   N   L   N   E   N
1741 gag atg agt gca gac cag cag gcc ttc caa gtg gcc atg agt gca gtg cag gag gct gag
 581  E   M   S   A   D   Q   Q   A   F   Q   V   A   M   S   A   V   Q   E   A   E
1801 gag gag gtg gtg gac ctg cac ggc cag tac ttt gcc cac aga gac aag atg gac aag atg
 601  E   E   V   V   D   L   H   G   Q   Y   F   A   H   R   D   K   M   D   K   M
1861 ctc atc cct ctg tac cag atg acc aac gaa gtg gat tat gac gtg gac gcc tat gcg cag
 621  L   I   P   L   Y   Q   M   T   N   E   V   D   Y   D   V   D   A   Y   A   Q
1921 cag ctt ggg gac gtg gtg ttg gaa aat ctg gag tgg tgg aat cag ctg agg gac cgc gtg
 641  Q   L   G   D   V   V   L   E   N   L   E   W   W   N   Q   L   R   D   R   V
1981 gtg aag ctg cgc cac cag ctg gag gaa gag gag aag ctg tcg cgt cgc cag cac gtg tga
 661  V   K   L   R   H   Q   L   E   E   E   E   K   L   S   R   R   Q   H   V   -
```

Figure S1. *Es-Kif2a* CDS full-length sequence clone. The CDS sequence and encoded amino acid sequence of *Es-Kif2a* are shown. atg (green), start codon; tga (red), stop codon. The CDS sequence of *Es-Kif2a* has been submitted to NCBI with the Genbank ID of ON014748.

A

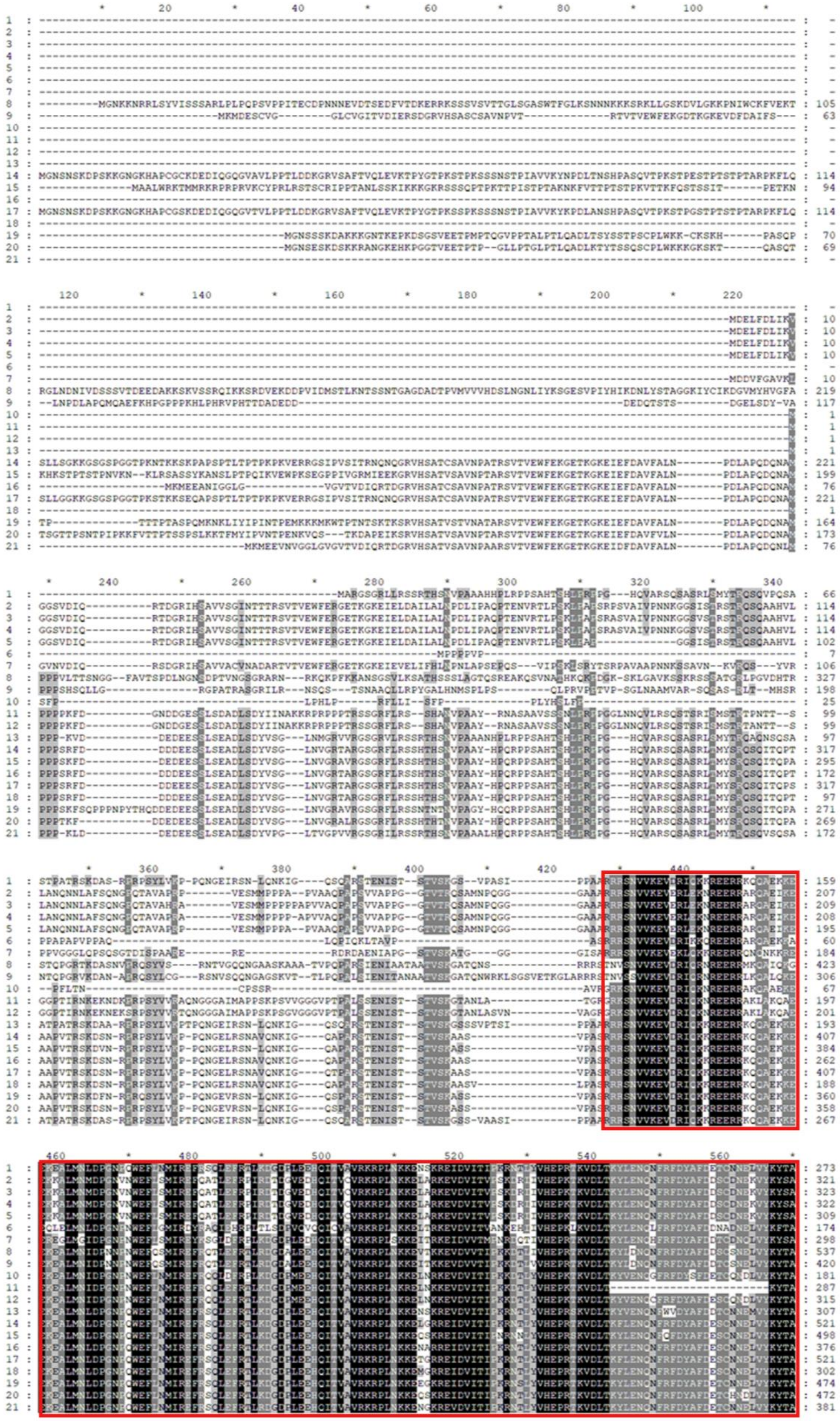

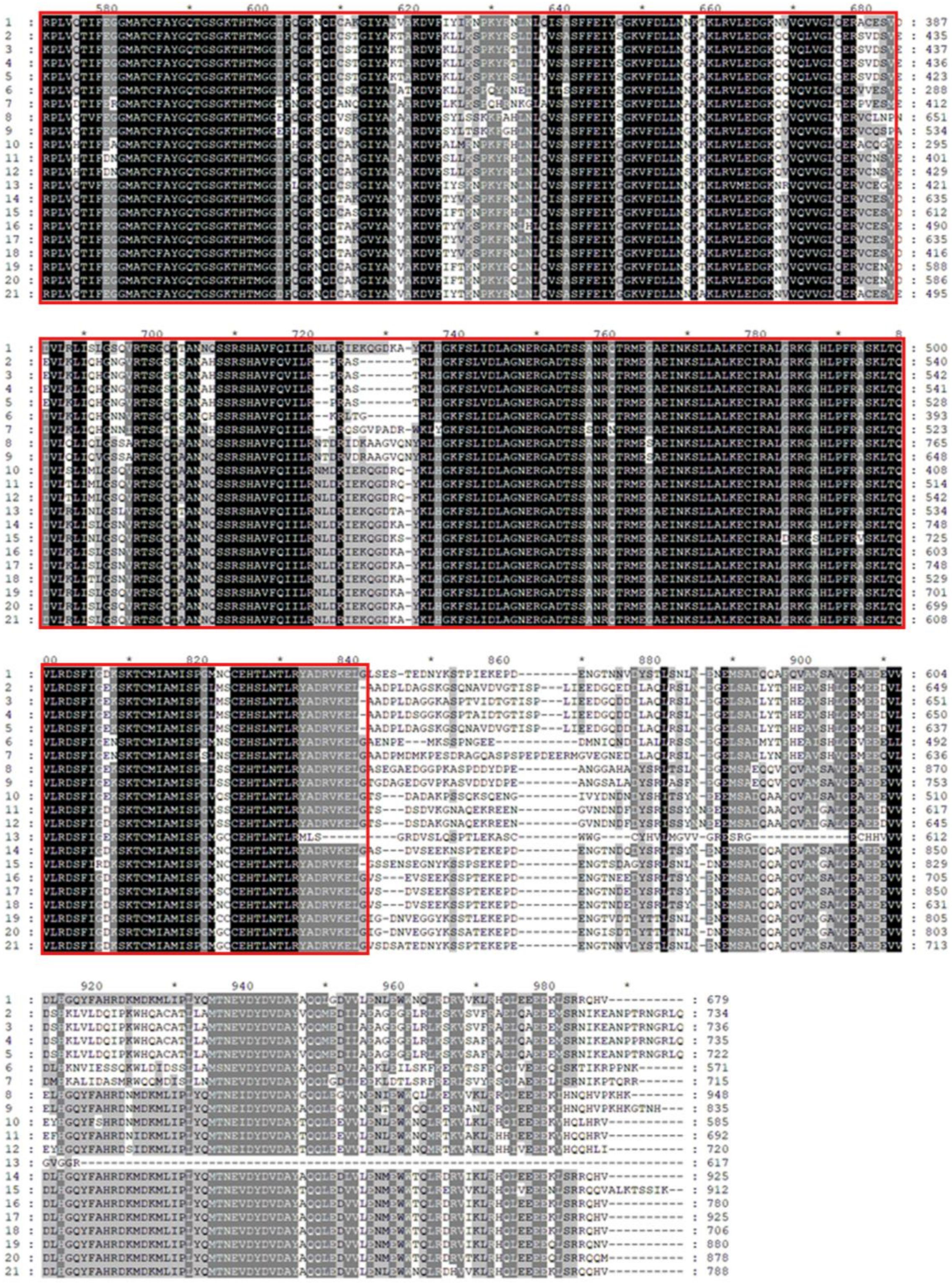

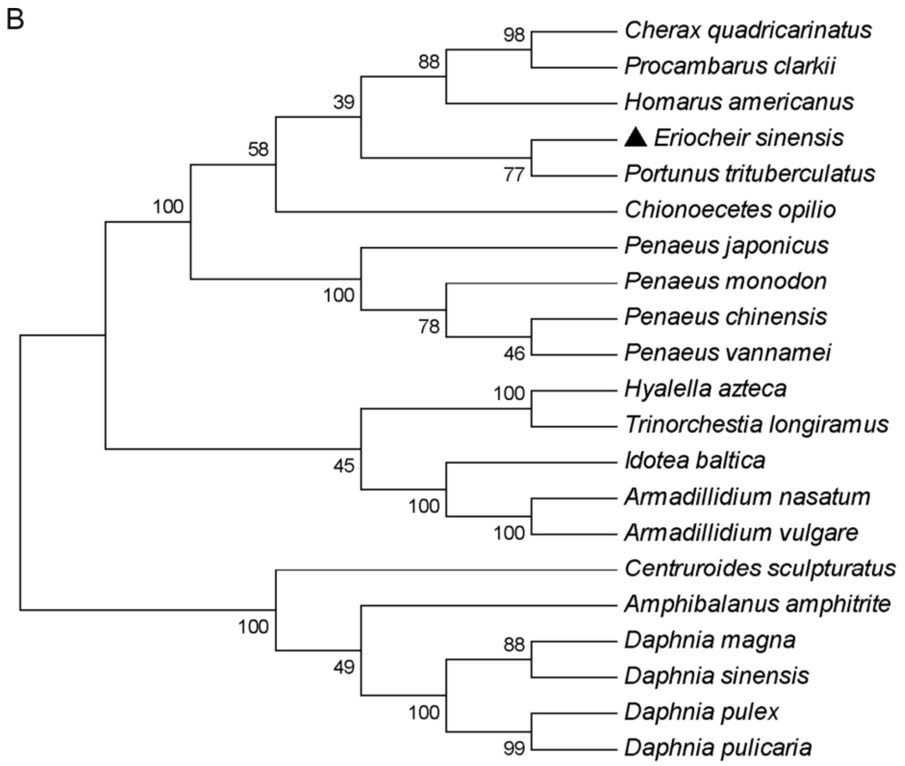

Figure S2. Multiple amino acid sequence alignment and evolutionary tree analysis of KIF2A proteins from different species. A. Multiple amino acid sequence alignment of KIF2A proteins from different species. Images were analyzed by MEGA 5 and GeneDoc software. Red boxes represent domains that are highly conserved across species. The species name and GenBank number of each homologous sequence are: 1. *Eriocheir sinensis* (ON014748); 2. *Daphnia pulicaria* (XP_046654477.1); 3. *Daphnia magna* (XP_032796177.2); 4. *Daphnia sinensis* (KAI9550034.1); 5. *Daphnia pulex* (EFX70810.1); 6. *Centruroides sculpturatus* (XP_023234851.1); 7. *Amphibalanus amphitrite* (XP_043237147.1); 8. *Trinorchestia longiramus* (KAF2368009.1); 9. *Hyalella azteca* (XP_018009240.1); 10. *Idotea baltica* (MCL4131360.1); 11. *Armadillidium nasatum* (KAB7499952.1); 12. *Armadillidium vulgare* (RXG69944.1); 13. *Chionoecetes opilio* (KAG0704647.1); 14. *Penaeus chinensis* (XP_047496930.1); 15. *Homarus americanus* (XP_042218487.1); 16. *Penaeus japonicus* (XP_042891107.1); 17. *Penaeus monodon* (XP_037778396.1); 18. *Penaeus vannamei* (XP_027217628.1); 19. *Procambarus clarkii* (XP_045594078.1); 20. *Cherax quadricarinatus* (XP_053636805.1); 21. *Portunus trituberculatus* (XP_045122812.1)。B. Phylogenetic tree analysis of KIF2A proteins from different species. The Neighbor-Joining method in MEGA5 software was used to construct an evolutionary tree for the above 21 species (bootstrap test, 1000 replicates).

```
   1 atg act agc cac gct aag gag gag ccc aag aag gag atc ccg gag att atc aag aac cct
   1  M   T   S   H   A   K   E   E   P   K   K   E   I   P   E   I   I   K   N   P
  61 tcc acg ggc gcc aaa tac cag cgg agt aaa ttc ttg ggc aag gga gga ttc gca aga tgt
  21  S   T   G   A   K   Y   Q   R   S   K   F   L   G   K   G   G   F   A   R   C
 121 tac gaa ctg aca gac atg aag acc aag gag ata ttt gcc ggg aag att gta ccc aag agc
  41  Y   E   L   T   D   M   K   T   K   E   I   F   A   G   K   I   V   P   K   S
 181 ctc ctc ctg aag ccc cac cag aag gag aag atg acc cag gag atc tgc atc cac cgc agc
  61  L   L   L   K   P   H   Q   K   E   K   M   T   Q   E   I   C   I   H   R   S
 241 ctc aag cac aag cac ctc gtc tcc ttc cat agc tac ttt gag gat ccc gat aac gtg tac
  81  L   K   H   K   H   L   V   S   F   H   S   Y   F   E   D   P   D   N   V   Y
 301 atc att ctg gag ctg tgc cgc agg agg tcc atg atg gag ctg cac aag agg cgg aag gcc
 101  I   I   L   E   L   C   R   R   R   S   M   M   E   L   H   K   R   R   K   A
 361 atc act gag tcc gag acg agg tac ttc ctg aag cag ctg ctg ctt ggt gtg aag cac ctg
 121  I   T   E   S   E   T   R   Y   F   L   K   Q   L   L   L   G   V   K   H   L
 421 cac gac aac aaa atc atc cac cgg gac ctg aag ctt ggc aac ctc ttc ctc aac gat gaa
 141  H   D   N   K   I   I   H   R   D   L   K   L   G   N   L   F   L   N   D   E
 481 atg gag ctc aag att ggc gac ttt ggt ctg gcc aca cgc atc gac tat gag ggg gag agg
 161  M   E   L   K   I   G   D   F   G   L   A   T   R   I   D   Y   E   G   E   R
 541 aag aga acc ctg tgt ggc act ccc aac tac att gct cct gag atc ctg tgc aag aag ggt
 181  K   R   T   L   C   G   T   P   N   Y   I   A   P   E   I   L   C   K   K   G
 601 cac agc tat gaa gtt gat gtc tgg tcc att ggt tgt atc ctg tat aca atg ttg gtg ggc
 201  H   S   Y   E   V   D   V   W   S   I   G   C   I   L   Y   T   M   L   V   G
 661 aag cct cca ttt gag acc cag aca ctg aag gac act tac ctg aga att aag aag aac gaa
 221  K   P   P   F   E   T   Q   T   L   K   D   T   Y   L   R   I   K   K   N   E
 721 tac cac atc ccc tcc cgc gtc ggc ccc ctc gct cgc tct ctc atc cag aaa ctg ctg cag
 241  Y   H   I   P   S   R   V   G   P   L   A   R   S   L   I   Q   K   L   L   Q
 781 gct gac cct ctg cag cgg ccc tgc gtc agc aag atc atc gaa gac gac ttc atg acc atg
 261  A   D   P   L   Q   R   P   C   V   S   K   I   I   E   D   D   F   M   T   M
 841 ggc tac atc ccc tcc cgg ctg ccc act tcc tgc ctc acc atg gcc ccg aga ttc gac tct
 281  G   Y   I   P   S   R   L   P   T   S   C   L   T   M   A   P   R   F   D   S
 901 cac tgc gct ctt gtg gcc acc agg aag cct ctt ctg gag atc aac aac aca gat ggt tct
 301  H   C   A   L   V   A   T   R   K   P   L   L   E   I   N   N   T   D   G   S
 961 cct gtg aag aac ata cag gag tgc ctg aag aag ggt gac agt gga ccc cgg ggt cac ccg
 321  P   V   K   N   I   Q   E   C   L   K   K   G   D   S   G   P   R   G   H   P
1021 cct tca ctt agt tct gcc agc aga ggg caa cag cag caa tca gca gca cac cag gaa gca
 341  P   S   L   S   S   A   S   R   G   Q   Q   Q   Q   S   A   A   H   Q   E   A
1081 gcc ccc cag cag ccc aag cac gat gaa ccc tca gac tgc tac ctc aag gac ctg tac aac
 361  A   P   Q   Q   P   K   H   D   E   P   S   D   C   Y   L   K   D   L   Y   N
1141 cag gtc aac tcc gtg gtc atg tcc aag cct gat gag cgg gac aac gtc aac gaa gag gag
 381  Q   V   N   S   V   V   M   S   K   P   D   E   R   D   N   V   N   E   E   E
1201 agc gaa gac ccg gca gca gta ccc atg atc tgg gtc agc aag tgg gtg gac tac agt gac
 401  S   E   D   P   A   A   V   P   M   I   W   V   S   K   W   V   D   Y   S   D
1261 aag tac ggc ctt ggc tac cag ctc tgt gat gac tcc atc ggt gtg ctc ttc aat gac ttc
 421  K   Y   G   L   G   Y   Q   L   C   D   D   S   I   G   V   L   F   N   D   F
1321 acc aaa ctg ttg ctg ctg gct gat gga gaa aac atc cac tac ata gag aga tcc aac ata
 441  T   K   L   L   L   L   A   D   G   E   N   I   H   Y   I   E   R   S   N   I
1381 gaa cat tac cat cag ctt aag aat tat cct gcg act ctc aac aag aag gta act ctt ctc
 461  E   H   Y   H   Q   L   K   N   Y   P   A   T   L   N   K   K   V   T   L   L
1441 cgc tac ttc cgc aac tac atg aac gaa cac ttg ttg aag gcc gct gca tcc atg tgc cca
 481  R   Y   F   R   N   Y   M   N   E   H   L   L   K   A   A   A   S   M   C   P
1501 cga gag gga gac gag ctg gca cgt atc ccc tcc ctc cgc acc tac ttc agg aca cgc tct
 501  R   E   G   D   E   L   A   R   I   P   S   L   R   T   Y   F   R   T   R   S
1561 gcc atc atc ctg cac ctc acc aac ggc acc ctc cag atc aac ttc ttt gag gat cac acc
 521  A   I   I   L   H   L   T   N   G   T   L   Q   I   N   F   F   E   D   H   T
1621 aag atc atc ctg tgc cct ctc atg ggt gct gtc act tac att gat gac aag agg aac ttc
 541  K   I   I   L   C   P   L   M   G   A   V   T   Y   I   D   D   K   R   N   F
1681 aag acc tac cga tta gcc agc ctg gag aag tat gga tgc cgg aaa gac tta gcc aac cgc
 561  K   T   Y   R   L   A   S   L   E   K   Y   G   C   R   K   D   L   A   N   R
1741 ctt cgt tat gcc cga aac atc atc cag aag ttg atg gtg acc aag tcc tct gtg aaa ccc
 581  L   R   Y   A   R   N   I   I   Q   K   L   M   V   T   K   S   S   V   K   P
1801 tcc acg tcc acc acc acc acc acc acc acc acc gtc acc tcc caa gag tcc cag ccc acc
 601  S   T   S   T   T   T   T   T   T   T   T   V   T   S   Q   E   S   Q   P   T
1861 aca gct tga
 621  T   A   -
```

Figure S3. *Es-Plk1* CDS full-length sequence clone. The CDS sequence and encoded amino acid sequence of *Es-Plk1* are shown. atg (green), start codon; tga (red), stop codon. The CDS sequence of *Es-Plk1* has been submitted to NCBI with the Genbank ID of OQ851420.

A

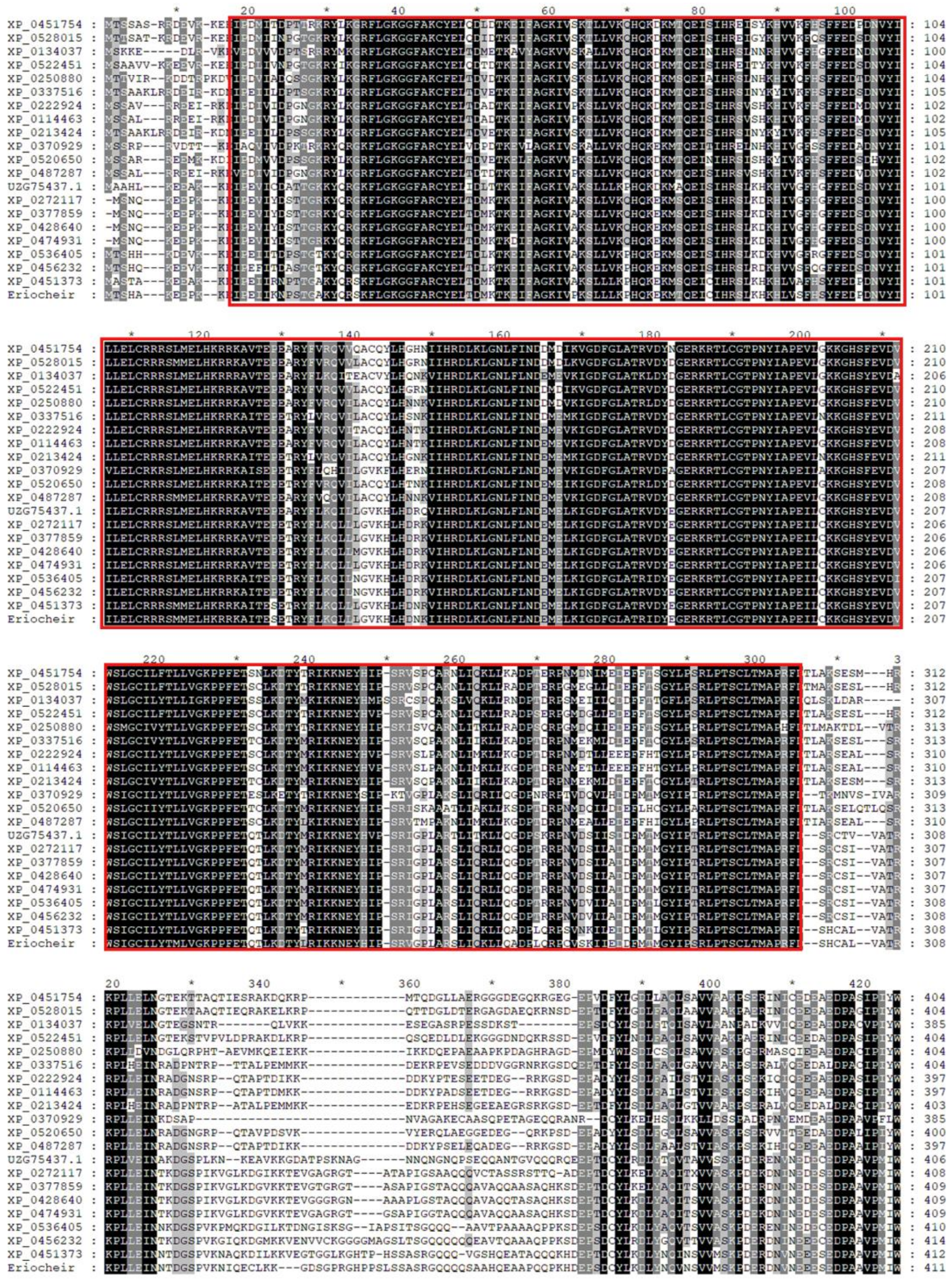

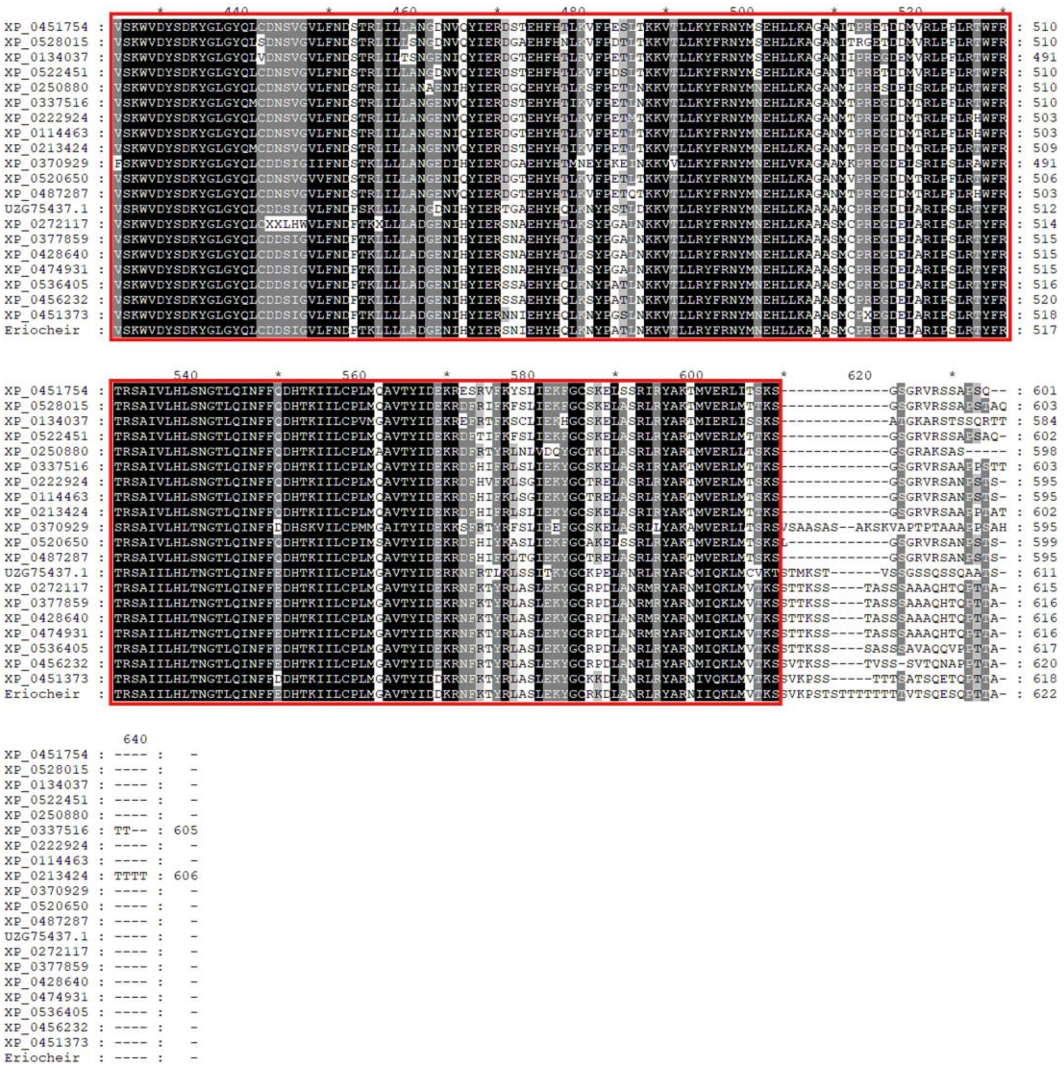

B

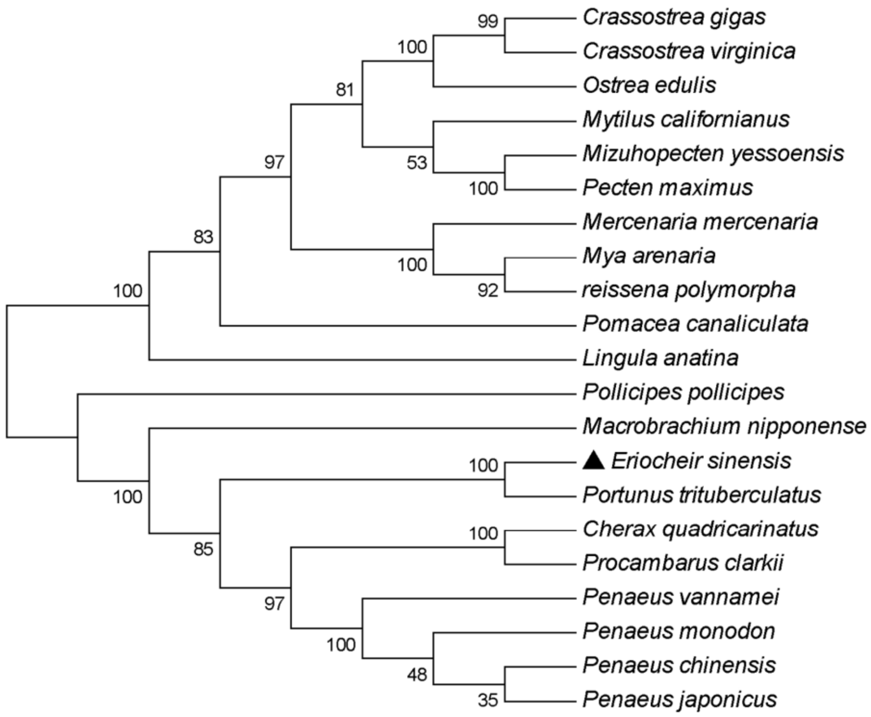

Figure S4. Multiple amino acid sequence alignment and evolutionary tree analysis of PLK1 proteins from different species. A. Multiple amino acid sequence alignment of PLK1 proteins from different species. Images were analyzed by MEGA 5 and GeneDoc software. Red boxes represent domains that are highly conserved across species. The species name and GenBank number of each homologous sequence are: 1. *Mercenaria mercenaria* (XP_045175493.2); 2. *Mya arenaria* (XP_052801532.1); 3. *Lingula anatine* (XP_013403756.1); 4. *Dreissena polymorpha* (XP_052245170.1); 5. *Pomacea canaliculata* (XP_025088006.1); 6. *Pecten maximus* (XP_033751629.1); 7. *Crassostrea virginica* (XP_022292480.1); 8. *Crassostrea gigas* (XP_011446350.1); 9. *Mizuhopecten yessoensis* (XP_021342443.1); 10. *Pollicipes pollicipes* (XP_037092997.1); 11. *Mytilus californianus* (XP_052065059.1); 12. *Ostrea edulis* (XP_048728781.1); 13. *Macrobrachium nipponense* (UZG75437.1); 14. *Penaeus vannamei* (XP_027211725.1); 15. *Penaeus monodon* (XP_037785985.1); 16. *Penaeus japonicus* (XP_042864001.1); 17. *Penaeus chinensis* (XP_047493190.1); 18. *Cherax quadricarinatus* (XP_053640533.1); 19. *Procambarus clarkii* (XP_045623220.1); 20. *Portunus trituberculatus* (XP_045137394.1); 21. *Eriocheir sinensis* (OQ851420). B. Phylogenetic tree analysis of PLK1 proteins from different species. The Neighbor-Joining method in MEGA5 software was used to construct an evolutionary tree for the above 21 species (bootstrap test, 1000 replicates).

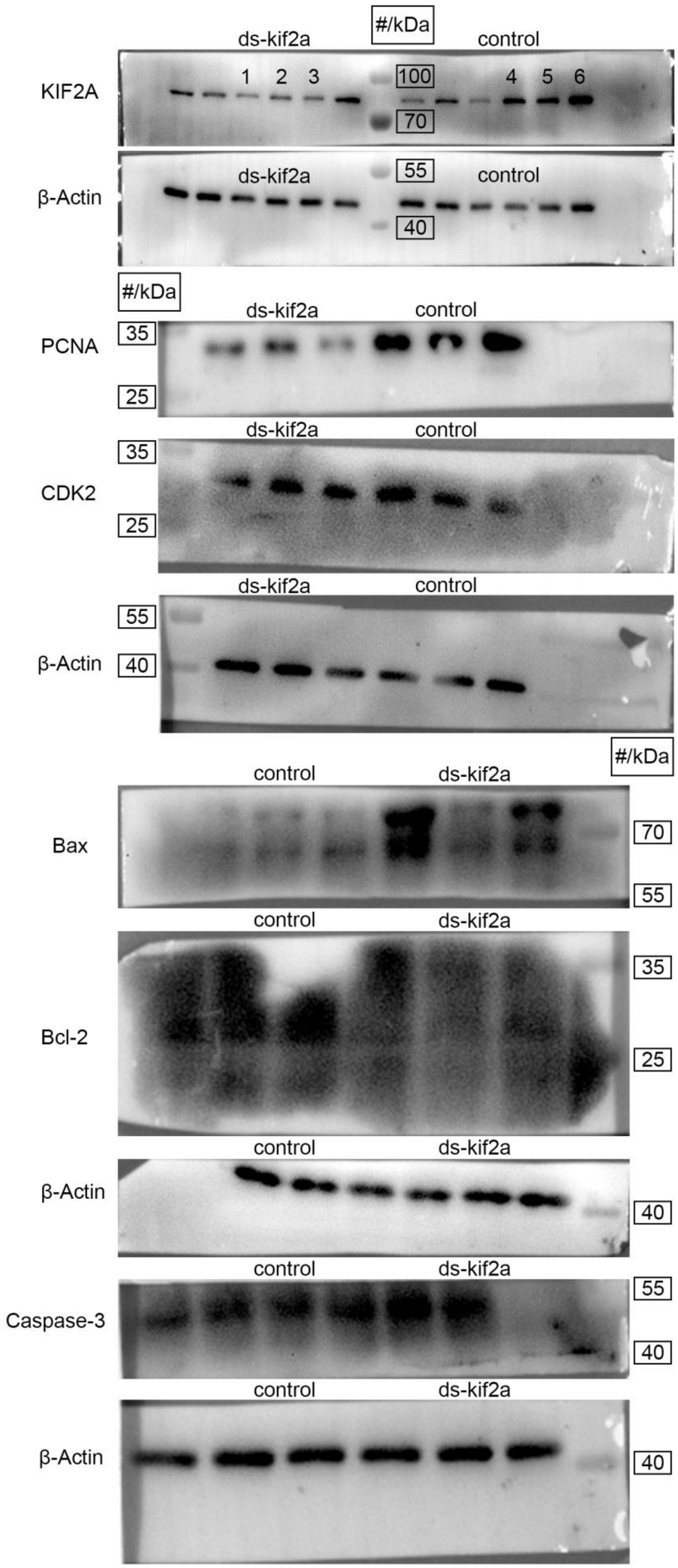

#/kDa
ds-kif2a
control
KIF2A
1
2
3
100
4
5
6
70
β-Actin
55
40
PCNA
35
25
CDK2
Bax
Bcl-2
Caspase-3

Figure S5. Western blot membranes of Es-KIF2A (~77 kDa), Es- PCNA (~29 kDa), Es-CDK2 (~34 kDa), Es-Bax (~58 kDa), Es-Bcl-2 (~30 kDa), Es-Caspase-3 (~49 kDa) in control and ds-Kif2a (*Es-Kif2a* interference) groups. The intensity ratios of KIF2A (KIF2A/β-actin) are 0.261674308, 0.347639599, 0.31803834, 0.728234938, 0.686466797, 0.946168845 from left to right (1~6). The intensity ratios of PCNA (PCNA/β-actin) are 1.160289103, 1.379899784, 0.967647572, 2.905533981, 2.237462451, 2.603057007 from left to right. The intensity ratios of CDK2 (CDK2/β-actin) are 0.69953079, 0.866453127, 1.070249738, 1.471553398, 1.615396365, 1.115534586 from left to right. The intensity ratios of Bax (Bax/β-actin) are 0.521788172, 0.651526531, 0.707675894, 1.013158633, 0.663387834, 0.751197605 from left to right. The intensity ratios of Bcl-2 (Bcl-2/β-actin) are 1.147134413, 1.380114286, 1.660770771, 0.626063064, 0.430404316, 0.669760479 from left to right. The intensity ratios of Caspase-3 (Caspase-3/β-actin) are 0.957175275, 0.962297049, 1.026981006, 1.160939095, 1.238976283, 1.211573362 from left to right. #Weight marker (molecular weight in kDa): Thermo Scientific Pierce Pre-Stained Protein Ladder, 10 to 170 kDa; catalogue number: 26616. Blot images were converted to grayscale with ImageJ as follows: Image -> Type –> 8 bit; Process->Subtract Background->Preview; Analysis->Set Measurements: Area, Mean gray value, Min & max gray value, integrated density; Analysis->Set Scale->Unit of length: pixels; Edit->Invert; Select the target band with a rectangle->Analysis->Measure.

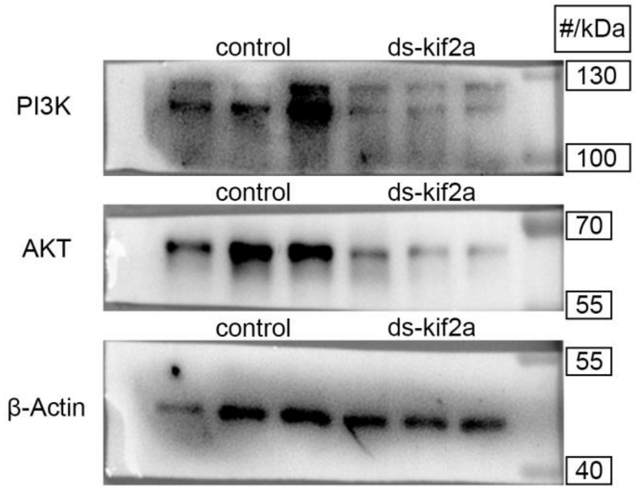

Figure S6. Western blot membranes of Es-PI3K (~110 kDa), Es-AKT (~56 kDa) proteins in control and ds-Kif2a (*Es-Kif2a* interference) groups. The intensity ratios of PI3K (PI3K/β-actin) are 0.731238688, 0.405939248, 0.568315158, 0.249210619, 0.147083716, 0.192286652 from left to right. The intensity ratios of AKT (AKT/β-actin) are 1.096022648, 1.082204355, 1.001434857, 0.573734066, 0.376028668, 0.266812546 from left to right. #Weight marker (molecular weight in kDa): Thermo Scientific Pierce Pre-Stained Protein Ladder, 10 to 170 kDa; catalogue number: 26616. Blot images were converted to grayscale with ImageJ as follows: Image -> Type –> 8 bit; Process->Subtract Background->Preview; Analysis->Set Measurements: Area, Mean gray value, Min & max gray value, integrated density; Analysis->Set Scale->Unit of length: pixels; Edit->Invert; Select the target band with a rectangle->Analysis->Measure.

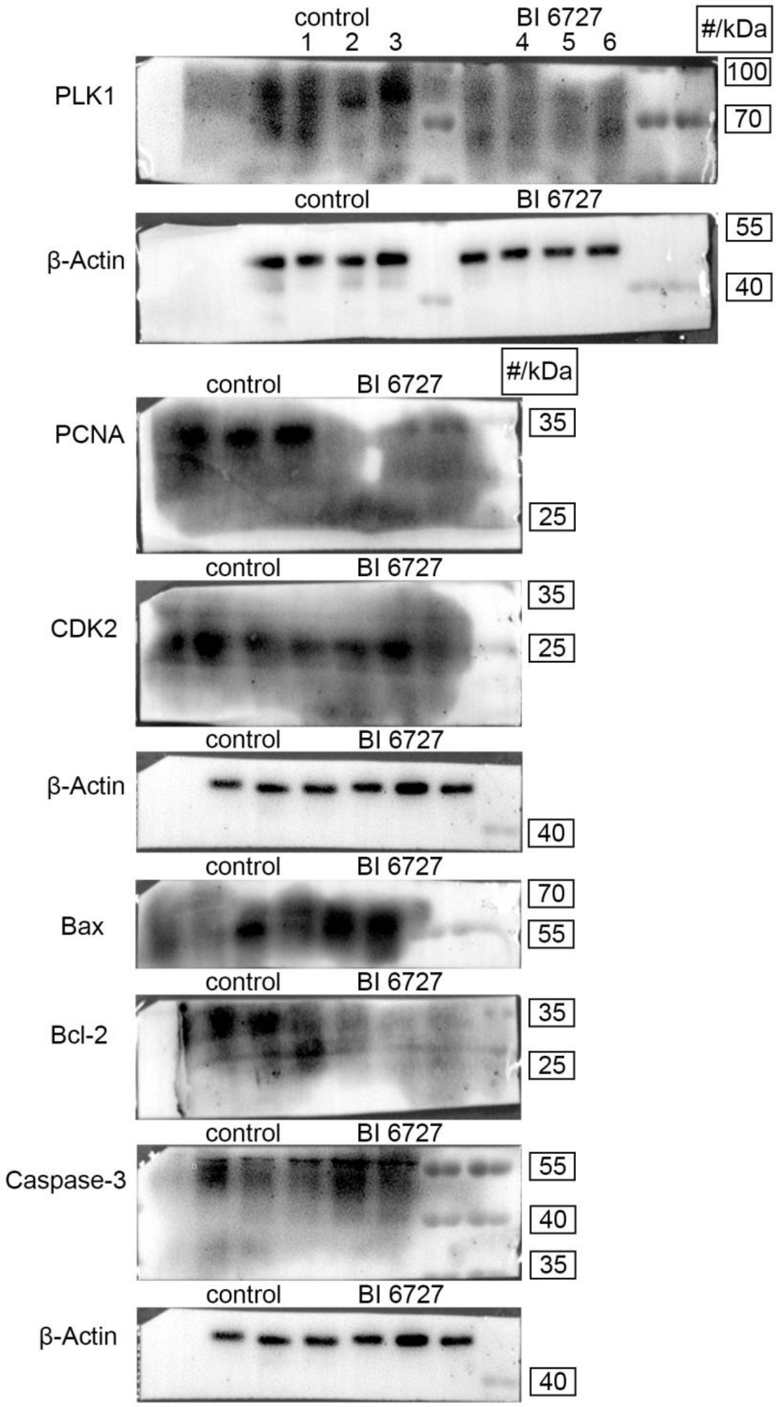

Figure S7. Western blot membranes of Es-PLK1 (~71kDa), Es- PCNA (~29 kDa), Es-CDK2 (~34 kDa), Es-Bax (~58 kDa), Es-Bcl-2 (~30 kDa), Es-Caspase-3 (~49 kDa) in control and BI 6727 (*Es-Plk1* inhibition) groups. The intensity ratios of PLK1 (PLK1/β-actin) are 1.10926509, 1.143473923, 1.028491177, 0.727195532, 0.770393432, 0.768009907 from left to right (1~6). The intensity ratios of PCNA (PCNA/β-actin) are 1.235370358, 0.968580295, 1.03160491, 0.584142747, 0.762964966, 0.804624322 from left to right. The intensity ratios of CDK2 (CDK2/β-actin) are 1.510206727, 1.647168348, 1.377385186, 1.029000415, 0.742847601, 1.078609615 from left to right. The intensity ratios of Bax (Bax/β-actin) are 0.900924214, 0.619607843, 1.126331702, 1.644797967, 1.593890979, 1.953162149 from left to right. The intensity ratios of Bcl-2 (Bcl-2/β-actin) are 1.78033396, 1.523273856, 1.243757409,

0.831477122, 0.777897934, 1.007276166 from left to right. The intensity ratios of Caspase-3 (Caspase-3/β-actin) are 0.068908906, 0.315748642, 0.253833505, 0.457379676, 0.382277636, 0.453013722 from left to right. #Weight marker (molecular weight in kDa): Thermo Scientific Pierce Pre-Stained Protein Ladder, 10 to 170 kDa; catalogue number: 26616. Blot images were converted to grayscale with ImageJ as follows: Image -> Type –> 8 bit; Process->Subtract Background->Preview; Analysis->Set Measurements: Area, Mean gray value, Min & max gray value, integrated density; Analysis->Set Scale->Unit of length: pixels; Edit->Invert; Select the target band with a rectangle->Analysis->Measure.

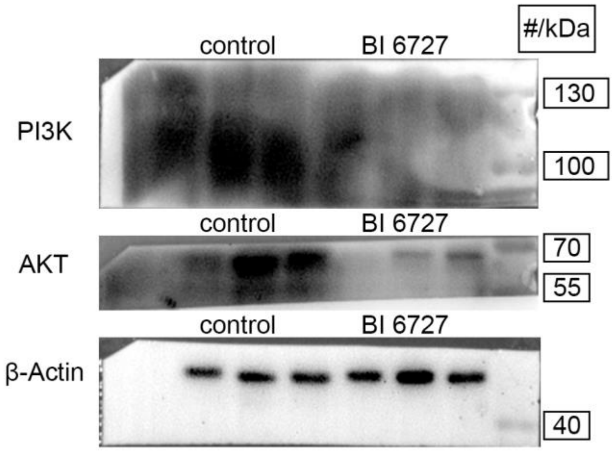

Figure S8. Western blot membranes of Es-PI3K (~110 kDa), Es-AKT (~56 kDa) proteins in control and BI 6727 (*Es-Plk1* inhibition) groups. The intensity ratios of PI3K (PI3K/β-actin) are 2.034442391, 1.765690921, 1.442034984, 1.124879122, 0.332578948, 0.417224478 from left to right. The intensity ratios of AKT (AKT/β-actin) are 0.790853909, 1.060318076, 0.95577607, 0.388696564, 0.375566546, 0.660818713 from left to right. #Weight marker (molecular weight in kDa): Thermo Scientific Pierce Pre-Stained Protein Ladder, 10 to 170 kDa; catalogue number: 26616. Blot images were converted to grayscale with ImageJ as follows: Image -> Type –> 8 bit; Process->Subtract Background->Preview; Analysis->Set Measurements: Area, Mean gray value, Min & max gray value, integrated density; Analysis->Set Scale->Unit of length: pixels; Edit->Invert; Select the target band with a rectangle->Analysis->Measure.

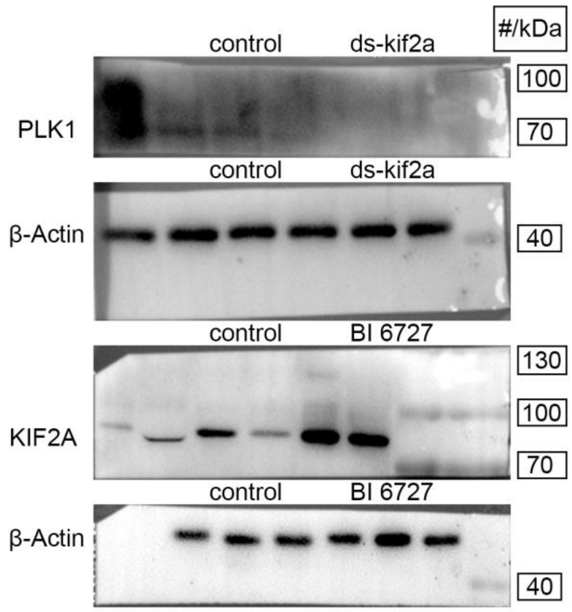

Figure S9. Western blot membranes of Es-PLK1 protein (~71 kDa) in control and ds-kif2a groups, and Es-KIF2A protein (~77 kDa) in control and BI 6727 groups. The intensity ratios of PLK1 (PLK1/β-actin) are 0.418122927, 0.294143012, 0.295493706, 0.20610752, 0.149084721, 0.171693735 from left to right. The intensity ratios of KIF2A (KIF2A/β-actin) are 0.232778805, 0.361955209, 0.61254679, 0.348736229, 0.804379431, 0.960495995 from left to right. #Weight marker (molecular weight in kDa): Thermo Scientific Pierce Pre-Stained Protein Ladder, 10 to 170 kDa; catalogue number: 26616. Blot images were converted to grayscale with ImageJ as follows: Image -> Type –> 8 bit; Process->Subtract Background->Preview; Analysis->Set Measurements: Area, Mean gray value, Min & max gray value, integrated density; Analysis->Set Scale->Unit of length: pixels; Edit->Invert; Select the target band with a rectangle->Analysis->Measure.
